# HIV incidence and sexual behavioral correlates among 4578 men who have sex with men (MSM) in Chengdu, China: a retrospective cohort study

**DOI:** 10.1186/s12889-021-10835-4

**Published:** 2021-04-26

**Authors:** Xinyi You, Stuart Gilmour, Wangnan Cao, Joseph Tak-fai Lau, Chun Hao, Jing Gu, Phuong Mai Le, Liping Peng, Dannuo Wei, Yang Deng, Xiaodong Wang, Huachun Zou, Jibin Li, Yuantao Hao, Jinghua Li

**Affiliations:** 1 School of Public Health, Sun Yat-sen University (North Campus), No.74, Zhongshan second road, Guangzhou, China; 2 Graduate School of Public Health, St. Luke’s International University, Tokyo, Japan; 3 Center for Evidence Synthesis in Health, School of Public Health, Brown University, Providence, RI USA; 4 Centre for Health Behaviours Research, JC School of Public Health and Primary Care, The Chinese University of Hong Kong, Hong Kong, China; 5 Sun Yat-sen Global Health Institute, Sun Yat-sen University, Guangzhou, China; 6 Chengdu Tongle Health Consulting Service Center, Chengdu, China; 7 School of Public Health (Shenzhen), Sun Yat-sen University, Shenzhen, China; 8 Department of Clinical Research, Sun Yat-sen University Cancer Center; State Key Laboratory of Oncology in South China; Collaborative Innovation Center for Cancer Medicine, Guangzhou, China

**Keywords:** HIV/AIDS, Incidence, Men who have sex with men, Cohort studies, Sexual behavior

## Abstract

**Background:**

The prevalence of HIV among men who have sex with men (MSM) in southwest China is still increasing. This study aimed to investigate the trend in HIV incidence and its associated risk factors among MSM in Chengdu, China.

**Method:**

Incidence data were collected from the largest local non-governmental organization (NGO) serving MSM in Chengdu between 2012 and 2018, while information on sexual behaviors was collected from 2014. All MSM who received voluntary counseling and testing services (VCT) in the collaborating NGO at least twice during the study period were included. We calculated the HIV incidence density among MSM every 2 years and the overall incidence rate. A Cox proportional hazards regression model was employed to identify risk factors for HIV infection.

**Result:**

A total of 4578 HIV-negative participants were included in the cohort. The total incidence density was 5.95 (95% *CI*: 5.37–6.56)/100 person-years (PYs) between 2012 and 2018. The segmented incidence density was 9.02 (95% *CI*: 7.46–10.78), 5.85 (95% *CI*: 4.86–6.97), 5.43 (95% *CI*: 4.53–6.46), and 3.09 (95% *CI*: 2.07–4.41)/100 PYs in 2012–2013, 2014–2015, 2016–2017, and 2018, respectively. After adjusting for sociodemographic characteristics, compared to participants without sexual partners within 6 months, MSM with one fixed partner (Adjusted Hazard Ratio, AHR = 1.18, 95% *CI*: 0.44–3.19) and more than five partners (AHR = 2.24, 95% *CI*: 0.81–6.20) had increased risk of HIV infection. MSM who used condom inconsistently had a higher risk of HIV infection (AHR = 1.87, 95% *CI*: 1.46–2.38) compared to consistent condom users.

**Conclusion:**

The decreased HIV incidence density among MSM was potentially related to the successful comprehensive HIV prevention strategies in Chengdu. Multiple male sexual partnerships and inconsistent condom use during anal intercourse were risk factors associated with HIV occurrence.

## Background

HIV/AIDS is a growing public health problem worldwide and in China, with a total of over 32 million people living with HIV/AIDS (PLWH) globally [1, 2]. The national statutory infectious diseases report showed that HIV/AIDS has always been the infectious disease with the highest rate of death in the past decade in China [3]. It is estimated that there were a total of 850,000 PLWH in the Chinese mainland by the end of 2018, accounting for 0.037% of the Chinese population [4, 5].

The national surveillance system reported that sexual transmission, especially male-to-male homosexual transmission, has become the major HIV transmission route in China [4, 6, 7]. The proportion of new HIV/AIDS cases reported yearly among MSM increased by more than 10 times in the last decade, from 2.5% in 2006 up to 28.3% in 2015 [8]. Studies have shown a rising trend in HIV prevalence among MSM, from 5.0% in 2009 to 6.9% in 2018 [9].

HIV incidence is the rate of new infections in a population in a specified time period and is a critical HIV surveillance indicator [10]. Calculation of HIV incidence is important to monitor trends in the HIV epidemic, and to evaluate interventions for HIV prevention [11]. However, compared to HIV prevalence studies, HIV incidence studies among Chinese MSM are limited, especially after 2015. Around 80 studies that reported HIV incidence rate/density among MSM in China have been identified, of which 12 were conducted in Sichuan and Chongqing (2006–2015), 12 were conducted in Jiangsu (2007–2015), and 14 were conducted in Beijing and Tianjin (2003–2015), but only one study [12] reported the incidence rate after 2015 (2008–2016) and only in Beijing. HIV incidence density is defined as the average incidence over a certain period of time. Its numerator is the number of persons newly diagnosed with HIV in the given period. The denominator of incidence density is the sum of person-time provided by observed participants [13]. The HIV incidence rate is defined as the number of new infections in the targeted population, usually expressed as a rate of infection per 100 persons per unit time [14]. Among these incidence studies, the majority used a repeated cross-sectional design [15], and only 30 studies used a cohort study design [8, 16]. The most updated meta-analysis calculated the pooled HIV incidence among Chinese MSM at 5.61/100 person years (PYs), with an increasing trend over time (5.29/100PYs in 2009–2011, 5.50/100PYs in 2012–2014) [17]. The pooled incidence value is the HIV incidence density calculated by meta-analysis [17]. The incidence density since 2015 among Chinese MSM is not well studied, even though testing and treatment strategies have expanded rapidly over that time period, and incidence studies are essential tools for understanding the impact of these strategies on the HIV epidemic [18].

The city of Chengdu has a population of more than 10 million urban residents and many migrant workers from other parts of China. Previous studies reported consistently high HIV incidence among MSM in Chengdu (8.92/100PYs, 95%*CI* = 7.38–10.46/100PYs) between 2011 and 2015, which is much higher than the number reported in other cities within Sichuan province (5.16/100PYs, 95%*CI* = 4.65–5.66/100PYs) and the national level (5.0/100PYs, 95%*CI* = 4.1–5.8/100PYs) [19, 20]. The situation after 2015 is unknown.

A series of prevention and control measures targeted at MSM were implemented in the 2010s by the local center for disease control and prevention (CDC) and non-governmental organizations in Chengdu [21–23], especially in the era of universal testing and antiretroviral treatment (ART). It is essential for policy makers to have an updated understanding of the HIV incidence trend and its risk correlates to better inform these prevention plans and programs [24].

This study aimed to update the trend in HIV incidence density among MSM in Chengdu until 2018, as well as to verify the association between sexual behavior-related factors and HIV seroconversion. Our findings will provide policy makers with evidence to propel sexual behavior-related prevention strategies to control the HIV epidemic among MSM.

## Methods

### Overall study design

A retrospective cohort study of trends in HIV incidence among MSM was conducted in Chengdu from 2012 to 2018, with the assistance of the Chengdu *Tongle* Health Consulting Service Center, which is the largest local gay-friendly non-governmental organization (NGO). This NGO is responsible for 80–90% of voluntary counseling and testing services (VCT) among MSM since its establishment in Chengdu. HIV testing was implemented by CDC doctors stationed at the NGO, and a total of 29,244 HIV tests were conducted from 2012 to 2018. Face-to-face interviews about sexual behaviors have been implemented since 2014 by NGO staff as a part of pre-test counselling. HIV testing and questionnaire results were linked with the participant’s mobile number, which was unique and used as the proxy for an identifier to match the HIV testing results to each participant. Participants’ mobile number was recoded into an unidentifiable ID number to make the dataset anonymous. Informed consent was obtained from all participants. HIV testing and the questionnaire survey were both routine activities of the NGO, and were not implemented as part of this research study.

### Study population and setting

The target population of our study was MSM who received VCT at the collaborating NGO and lived in Chengdu at the time of the test. Eligible criteria for study enrollment included: 1) aged 16 years and above; 2) males who ever had oral or anal sex with other male(s) during their lifetime; 3) self-reported serological status was HIV-negative or unknown at the study enrollment; and 4) had at least two HIV testing records in the collaborating NGO during the study period.

### Questionnaire interview

The questionnaire included two parts. The first part collected information on sociodemographic characteristics such as age, residential district, education, marital status and mobile number. The second part collected information on sexual behaviors in the past 6 months. Questions on sexual behaviors included number of fixed, casual or commercial male sexual partners, primary venue to find sexual partners (e.g., bars, bathhouses, parks/public toilets, online chat rooms, BBS, mobile dating apps), and frequency of condom use (i.e., never, sometimes, every time).

### Statistical analysis

Descriptive analyses were conducted to show the sociodemographic characteristics of participants. Categorical variables were reported as both number and percentage, while continuous variables were presented as “mean ± standard deviation”. The midpoint between the date of the last negative and the first positive test was assumed to be the onset date of HIV infection in our analysis. HIV incidence density and its 95% confidence interval (95%*CI*) was calculated with person-years (PYs) over the observed time as the denominator, assuming a Poisson distribution [25, 26]. Chi-square tests were used to compare behavioral characteristics between participants with positive and negative HIV serostatus. Marginally significant variables (*p* ≤ 0.10) in univariate analysis were included in a multivariate Cox proportional hazards regression model to identify risk behavior factors associated with HIV incidence [27] after adjusting for sociodemographic characteristics. All analyses were performed using both IBM SPSS software Version 25.0 (SPSS Inc., Chicago, IL USA) and SAS Version 9.4 (Cary, North Carolina, USA). A two-tailed *p* < 0.05 was considered to be statistically significant.

## Results

### Demographic characteristics

**Fig. 1:**
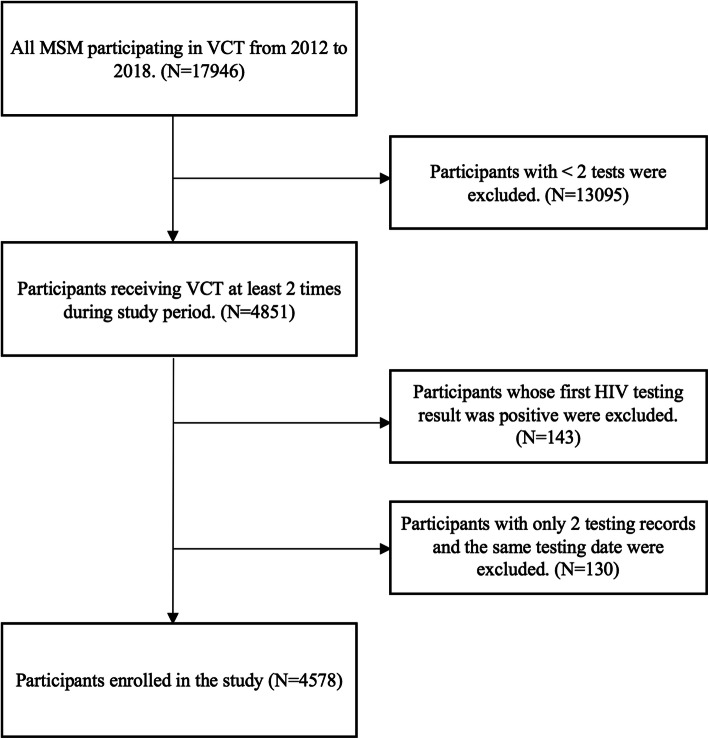
Retrospective cohort study screening process, 2012–2018

A total of 4578 HIV-negative MSM with at least two HIV testing records were identified and included in our cohort (Fig. 1). Of 4578 HIV-negative MSM who were enrolled in this retrospective cohort study, 3168 (69.2%) MSM were younger than 30 years old, and 1066 (23.3%) were between 31 and 45 years old. Two thirds (64.9%) of the participants had a college degree or above. The majority (76.8%) of the participants were single when enrolled at baseline (Table 1).

**Table 1:** Participants’ baseline characteristics between 2012 and 2018

Characteristics	N (%)
**Age (years old)** ^a^	
≤ 30	3168 (69.2)
30–45	1066 (23.3)
46–59	287 (6.3)
≥ 60	57 (1.2)
**Education** ^a^	
Junior high school or less	573 (12.5)
Senior high school/secondary technical school	1036 (22.6)
College or higher	2969 (64.9)
**Marital status** ^a^	
Single	3517 (76.8)
Married with a woman	842 (18.4)
Divorced/widowed	219 (4.8)
**Number of male sexual partners in the past 6 months** ^b^	
Zero	250 (7.0)
One fixed male sexual partner	1140 (31.9)
Multiple male sexual partners	2189 (61.2)
**Primary venue to find sexual partners** ^b^	
No male sexual partners	222 (6.2)
Offline	745 (20.8)
Online	1449 (40.5)
Money boys from clubs	1074 (30.0)
Other venues	89 (2.5)
**Condom use during anal intercourse in the past 6 months** ^b^	
Never	225 (6.3)
Sometimes	1352 (37.8)
Every time	1575 (44.0)
No anal intercourse	427 (11.9)

^a^ The baseline characteristics data were collected since 2012 (*N* = 4578)

^b^ These baseline characteristics data were collected since 2014 (*N* = 3579)

### Sexual behaviors

Among 3579 MSM (enrolled after 2014) with information on sexual behaviors, 7.0% had no partners in the past 6 months, 31.9% had one partner, and 61.2% had two or more male sexual partners. The primary venue to find sexual partners was through online apps (40.5%), followed by money boys from clubs (30.0%) and offline (20.8%). Less than half (44.0%) of the participants reported consistent condom use during anal intercourse in the past 6 months while 6.3% never used a condom (Table 1).

### HIV incidence density

**Fig. 2:**
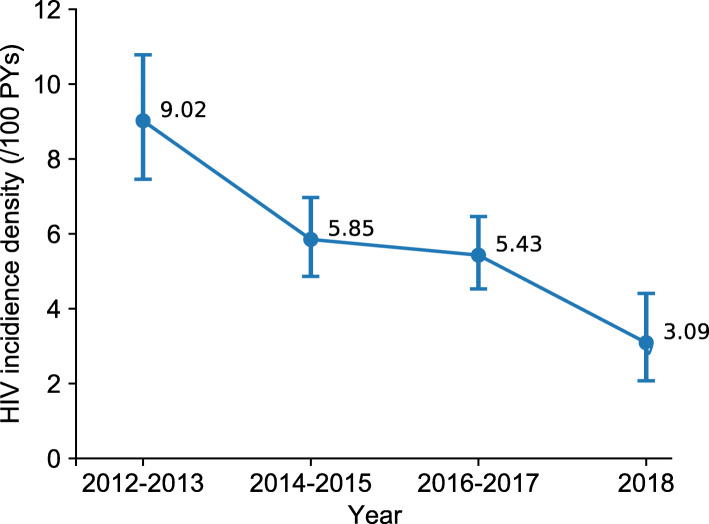
Trend in HIV incidence density and 95% confidence interval among Chengdu MSM from 2012 to 2018

Between 2012 and 2018, a total of 381 HIV seroconversions were detected with 6406 person-years (PYs) observed. The overall HIV incidence density was 5.95 per 100 PYs (95%*CI*: 5.37–6.56) (Table 2). The segmented incidence density was 9.02 (95%*CI*: 7.46–10.78), 5.85 (95%*CI*: 4.86–6.97), 5.43 (95%*CI*: 4.53–6.46), and 3.09 (95%*CI*: 2.07–4.41) per 100 PYs in 2012–2013, 2014–2015, 2016–2017, and 2018 respectively. The HIV incidence density among MSM showed a decreasing trend since 2012 (Fig. 2).

**Table 2:** HIV incidence density of MSM in Chengdu between 2012 and 2018

Year	Number of Seroconversions, n	Observed person-years (PYs)	Incidence Per 100 PYs	95% ***CI***
2012–2013	113	1253	9.02	(7.46–10.78)
2014–2015	119	2034	5.85	(4.86–6.97)
2016–2017	122	2245	5.43	(4.53–6.46)
2018	27	874	3.09	(2.07–4.41)
Total	381	6406	5.95	(5.37–6.56)

### Sexual behaviors associated with HIV incidence

The variable measuring primary method of finding sexual partners was not statistically significant in the univariate and multivariate regression analyses, and was excluded from the final model. Multiple male sexual partners and inconsistent condom use were both risk factors for HIV incidence in the multivariable analysis. After adjusting for sociodemographic characteristics, compared to participants without sexual partners within the last 6 months, MSM with one fixed partner (Adjusted Hazard Ratio, AHR = 1.18, 95%*CI*: 0.44–3.19), with two to five sexual partners (AHR = 1.51, 95%*CI*: 0.56–4.05) or with more than five partners (AHR = 2.24, 95%*CI*: 0.81–6.20) had increased risk of HIV infection. MSM who used condom inconsistently during anal intercourse had higher risk of HIV infection (AHR = 1.87, 95%*CI*: 1.46–2.38) compared to consistent condom users (Table 3).

**Table 3:** Sexual behaviors associated with HIV incidence among participants

Characteristics	Number of Seroconversion, n (row %) ***N*** = 288 (8.0%)	Unadjusted HR ^a^ (95%***CI***)	Adjusted HR ^a^ (95% ***CI***)	***p*** value
**Age**				0.244
≤ 30	157 (6.6)	1.00	1.00	
30–45	89 (10.1)	1.17 (0.90–1.52)	0.91 (0.66–1.27)	0.594
46–59	32 (11.5)	1.23 (0.84–1.80)	0.88 (0.55–1.42)	0.606
≥ 60	10 (27.8)	2.67 (1.41–5.08)	1.76 (0.85–3.61)	0.126
**Education**				< 0.001
Junior high school or less	61 (15.4)	2.31 (1.72–3.11)	1.95 (1.40–2.73)	< 0.001
Senior high school/secondary technical school	65 (9.4)	1.36 (1.02–1.81)	1.31 (0.97–1.76)	0.080
College or higher	162 (6.5)	1.00	1.00	
**Marital status**				0.416
Single	186 (6.9)	1.00	1.00	
Married	69 (10.2)	1.24 (0.94–1.63)	0.98 (0.67–1.44)	0.932
Divorced/widowed	33 (16.1)	1.71 (1.18–2.49)	1.29 (0.82–2.03)	0.275
**Number of male sexual partners in the past 6 months** ^b^				0.001
0	7 (2.8)	1.00	1.00	
1	76 (6.7)	2.35 (1.08–5.09)	1.61 (0.73–3.55)	0.239
2–5	169 (8.9)	3.11 (1.46–6.62)	2.18 (1.01–4.72)	0.047
> 5	36 (12.6)	4.89 (2.18–10.99)	3.19 (1.40–7.26)	0.006
**Primary venue to find sexual partners** ^b^			N.S.	
No male sexual partners	14 (6.3)	1.00		
Offline	79 (10.6)	1.75 (0.99–3.09)		
Online	109 (7.5)	1.59 (0.91–2.78)		
Money boys from clubs	84 (7.8)	1.53 (0.87–2.69)		
Other venues	2 (2.2)	0.59 (0.13–2.61)		
**Condom use during anal intercourse in the past 6 months** ^b^				< 0.001
Inconsistent	174 (11.0)	2.04 (1.61–2.59)	1.87 (1.46–2.38)	< 0.001
Consistent	114 (5.7)	1.00	1.00	

^a^*HR* Hazard Risk

^b^ These characteristics data were collected since 2014 (N = 3579)

## Discussion

The study found that the incidence of HIV among MSM in Chengdu, a first-line megacity in southwest China, showed a declining trend from 2012 to 2018, but still remained at a high level. The continuous decrease in HIV incidence suggested good progress in HIV prevention measures in Chengdu, but the overall high HIV incidence density suggested that MSM are still at high risk of HIV infection.

HIV incidence identified in this study was slightly higher, compared to the meta-analysis result of HIV incidence among MSM in China (5.0/100PYs) published in 2015 [19]. MSM in Chengdu remain a vulnerable subpopulation for HIV infection, and should be considered as a priority for HIV/AIDS intervention projects. There are ongoing HIV control efforts among MSM in Chengdu, including enhanced VCT, universal ART, condom promotion, and other behavioral interventions. More prevention options and efforts should be made available to the high-risk group of MSM, such as pre-exposure prophylaxis (PrEP).

Previous studies conducted in some megacities/provinces have also shown high HIV incidence among MSM, for example, 5.16% in Sichuan in southwest China from 2011 to 2015 and 6.0/100PYs to 7.8/100PYs in Beijing during 2012 and 2015 [28, 29]. A cohort study in Hangzhou, a developed second-line city in eastern China, reported an HIV incidence of 6.6/100PYs among MSM between 2014 and 2015 [16]. The segmented incidence density in this time period among MSM in Chengdu was 5.85/100PYs, which was lower than some other first-line and second-line cities in China.

The declining trend in HIV incidence identified in this study was similar to trends observed globally. The overall rate of HIV incidence among MSM in Chengdu was higher than the rate in the USA during 2009–2015 (4.11/100PYs) [27], and also higher than the rate in India (0.87/100PYs) [27] in 2012, and in Australia (0.048/100PYs) [30] in 2016. Higher rates have been observed in Myanmar in 2016 with 10.1/100PYs [27], followed by four African countries in 2017 with 6.96/100PYs [15]. Total time under observation among MSM in this study was larger compared to similar studies in Australia, the USA and the Netherlands [31] (with 4100, 632 and 171 person-years respectively), suggesting that this incidence study is one of the largest and most robust studies to have been conducted in a single city globally, offering high precision in its estimates and trends.

The current study found that multiple male sexual partners and inconsistent condom use are risk factors of HIV incidence among MSM, which are consistent with many existing studies [32]. We conducted additional analysis to explore the changes in HIV-related behaviors in this sample of the MSM population. The results showed significant changes in sexual behaviors over time among this open cohort of MSM in Chengdu from 2014 to 2018. The rate of condom use increased from 42.4% in 2014 to 55.8% in 2018 (trend test *p* < 0.001). In addition, the lifetime HIV testing rate also increased from 51.2% in 2014 to 75.2% in 2018 (trend test *p* < 0.001). This is consistent with the trend in HIV incidence density among MSM in Chengdu. Therefore, we conclude that improved sexual behaviors and HIV testing may have partially contributed to the reduction of HIV incidence density in Chengdu. Furthermore, our study found about 30% of MSM in Chengdu obtained their primary sex from money boys in clubs, who offer commercial sex services to men. In the twentieth century, there was a large migration from rural areas to urban areas in southwest China. Many young men in their twenties have increasingly entered the sex trade, and are known as money boys [33]. This subgroup is six times more likely to be infected with HIV than other MSM in Chengdu [34]. In line with another studies, we considered that money boys and their clients may act as bridging populations for HIV transmission from high-risk MSM to low-risk MSM [35]. These susceptible subpopulations among MSM require more efforts in implementing both behavioral and biomedical interventions. Consistent with previous studies in China, we founded that inconsistent condom use remained the most easily identifiable risk factor for HIV incidence in this study [12, 36, 37]. One study conducted in Beijing revealed that increased risk of HIV infection was strongly associated with lower level of perceived social norms about condom use [38]. It shows the continuing importance of behavioral interventions for HIV prevention among Chinese MSM, even in an era of high rates of testing and treatment. Unprotected anal intercourse (UAI) is considered the most prominent risk factor for HIV infection not only in China but worldwide [39, 40]. Some researchers suggested that feelings of trust, desired intimacy and pleasure could lead MSM to not use condoms during anal sex [37]. Therefore, the guidelines on HIV prevention for key populations published by WHO in 2014 have highlighted that increasing the availability, accessibility, affordability and use of condoms among MSM is an essential component of the HIV response [41].

Effective prevention strategies are always the key to decrease new HIV infections among MSM. In addition to advancing promotion of behavioral change interventions such as condom use among MSM in response to the WHO recommendations, other biomedical interventions such as HIV testing and ART have also been promoted in China during the past 10 years [42]. Several modeling studies using the Asian Epidemic Model and compartmental models indicated that condom use in combination with ART could substantially reduce HIV incidence among MSM in China [43–45]. In China, ART coverage also benefits from increased VCT services [43]. Another modeling study conducted in Guangzhou, China concluded that the increase of HIV incidence had been curbed since the AIDS comprehensive Prevention project which was launched in 2010 to improve condom use, HIV testing and ART coverage [46].

This study is subject to several limitations. First, the result may be biased due to the convenience-sampling method. Participants included in our study were active service users of a gay-friendly NGO, and they tend to be younger and highly educated. Findings of this study might not be generalizable to other MSM populations, such as non-VCT users and MSM in other cities. Despite the relatively large sample size, we included only those with two or more HIV testing records at the NGO during the study period. Second, reporting bias may exist due to the nature of self-reported data and the sensitive questions about sexual behaviors. Third, except for sexual behaviors, other factors (e.g. drug use) associated with HIV incidence among MSM were under-investigated in the present study.

## Conclusions

The incidence density of HIV among MSM decreased gradually from 2012 to 2018, which was potentially related to the successful comprehensive HIV prevention strategies in Chengdu. Multiple male sexual partnerships and inconsistent condom use continue to be risk factors associated with HIV occurrence, which highlights the need for promoting HIV/AIDS-related knowledge, enhanced behavioral interventions, and strengthening related services for vulnerable populations.

## Data Availability

The data used in the study was not publicly available. The datasets used and analyzed during the current study are available from the corresponding author on reasonable request.

## Abbreviations

MSM: Men who have sex with men
NGO: Non-governmental organization
VCT: Voluntary counseling and testing services
PYs: Person-years
AHR: Adjusted Hazard Ratio
PLWH: People living with HIV/AIDS
CDC: Center for disease control and prevention
ART: Antiretroviral treatment
PrEP: Pre-exposure prophylaxis
